# Editorial: Higher education and non-cognitive skill development: Why, what and how?

**DOI:** 10.3389/fpsyg.2022.1000725

**Published:** 2022-09-14

**Authors:** Paula Alvarez-Huerta, Angie L. Miller, Inaki Larrea, Alexander Muela

**Affiliations:** ^1^Innovation and Intervention in Inclusive Education, HUHEZI, Mondragon Unibertsitatea, Eskoriatza, Spain; ^2^Center for Postsecondary Research, Indiana University School of Education, Bloomington, IN, United States; ^3^Department of Clinical and Health Psychology and Research Methodology, University of the Basque Country UPV/EHU, Donostia, Spain

**Keywords:** higher education, productivity in research scholarship (PQ), basic education, non-cognitive skill, critical thinking, innovation, creativity

Students will need more than knowledge in a society characterized by dynamism and unpredictability. Preparing graduates for such an uncertain scenario remains a challenge to higher education institutions. In this regard, the need to promote specific skills and behaviors through educational models based on the development of competences is highlighted (García, 2016). It is argued that in addition to subject-specific knowledge, students will need critical thinking, creativity, innovation, and resilience, as well as empathy, sense of responsibility, and the ability to collaborate with others. Accordingly, incorporating these non-cognitive skills into existing curricula and pedagogy is on the agenda of higher education institutions across the world.

Nevertheless, most research on the student non-cognitive development has been conducted at the elementary and secondary school level, and there is a need to promote the development of non-cognitive skills at the higher education level (Schonert-Reichl, 2019). Although evidence seems to indicate that higher education has the potential to promote key learning outcomes among students (Kassenboehmer et al., 2018), there is scarce critical evidence on what these competence-based educational models entail and little consensus on what should be measured and how. Furthermore, few studies elaborate on which specific learning environments shape such skills.

As a common theme, this special issue focuses on analyzing and understanding the university student experience and on identifying key themes for promoting student integral development. Drawing on different theoretical backgrounds, these articles present elements for reflection on how to improve the experience of university students and prepare them for the future of work.

In the first of the studies that make up this special issue, Ahuja et al. presents a case study of a university in which student non-cognitive development is an explicit objective reflected in structures, programs, and curricula. Although this framework may not be directly replicable in other higher education institutions, it does offer an innovative approach to the development of skills such as self-awareness or critical thinking, central to the future development of coming generations.

Students commence university with different socioeconomic and personal characteristics, and also unique life experiences. Acknowledging this ought to be the first step to improve the quality of the academic programs offered by higher education institutions. In their study, Smith et al. analyze the different factors that affect success among domestic and international students. Psychological needs, social relationships and connections to campus, and learning preferences and behaviors are some of the factors to take into account when analyzing the development of the different groups of students, especially in relation to minorities, which according to the literature, might have more trouble adjusting to the college experience (Carter, 2006; Llamas et al., 2020).

According to Caballero-Garcia and Sanchez-Ruiz, analyzing student adjustment is relevant as it can affect not only student university experiences but also their future academic performance, productivity, and excellence in the working environment. Specifically, in their study they highlight the importance of building learning environments in which creativity and life satisfaction of students are encouraged. Despite calls to the need to promote creativity in higher education (Badger, 2019), there remains significant work to be done (Grigorenko, 2019), particularly with regard to acquiring empirical evidence on those learning contexts that might promote creativity among coming generations (Marquis et al., 2017).

If allocating resources to promoting the non-cognitive skills of university students is still a challenge in higher education, it is even more so at the postgraduate level (Le Roux, 2018). Despite the evidence pointing to mental health issues and low levels of retention among postgraduates (Hazell et al., 2020), strategies aimed at improving postgraduate student integral development are not prioritized in most university departments. The review by Frantz et al. highlights that despite the low occurrence of such strategies, the literature on non-cognitive skill development among postgraduate students is auspiciously growing. In their study, they also indicate the need to clarify the skills necessary to thrive in the postgraduate context, as well as the need to improve their conceptualization.

The identification of key competencies required for the success of PhD candidates is the objective of the study carried out by Lasekan et al. The use of an innovative framework, based on contemporary cultural references, allows the identification of central competencies for the career of young researchers. In addition, their study offers specific tools and strategies to support these competencies at the tertiary level.

Each of these articles is reflective of the need to rethink educational models. Progressing from traditional higher education outcomes, such as knowledge acquisition or occupational success, requires empirical evidence and collective discussion about those skills and educational strategies that will allow the student to contribute to social progress. Moreover, the pandemic has had a significant impact on the wellbeing of university students (Marques et al., 2021; Pinals, 2021), but it is also an opportunity to reflect on the role that educational institutions can play in nurturing student psychological, emotional, and wellbeing needs. It is important to note that each of these studies have been carried out in different international contexts, a call to the importance of having a global perspective when analyzing student development without overlooking student unique cultural backgrounds.

## Author contributions

All authors worked on the Research Topic and contributed to the article and approved the submitted version.

## Conflict of interest

The authors declare that the research was conducted in the absence of any commercial or financial relationships that could be construed as a potential conflict of interest.

## Publisher's note

All claims expressed in this article are solely those of the authors and do not necessarily represent those of their affiliated organizations, or those of the publisher, the editors and the reviewers. Any product that may be evaluated in this article, or claim that may be made by its manufacturer, is not guaranteed or endorsed by the publisher.
